# Early peroxisome proliferator-activated receptor gamma regulated genes involved in expansion of pancreatic beta cell mass

**DOI:** 10.1186/1755-8794-4-86

**Published:** 2011-12-30

**Authors:** Yurena Vivas, Cristina Martínez-García, Adriana Izquierdo, Francisco Garcia-Garcia, Sergio Callejas, Ismael Velasco, Mark Campbell, Manuel Ros, Ana Dopazo, Joaquin Dopazo, Antonio Vidal-Puig, Gema Medina-Gomez

**Affiliations:** 1 Universidad Rey Juan Carlos. Dpto. de Bioquímica, Fisiología y Genética Molecular. Avda.de Atenas s/n. 28922. Alcorcón. Madrid. Spain; 2 Functional Genomics Node, National Institute for Bioinformatics. Centro de Investigacion Prıncipe Felipe, Camino de las Moreras, 46012 Valencia, Spain; 3 Genomics Unit. CNIC (Centro Nacional de Investigaciones Cardiovasculares). Fernández Almagro, 3. 28029 Madrid, Spain; 4 University of Cambridge Metabolic Research Laboratories. Institute of Metabolic Science, NIHR Cambridge Biomedical Research Centre Level 4. Addenbrooke's Hospital, Hills Rd. Cambridge CB2 OQQ. UK

## Abstract

**Background:**

The progression towards type 2 diabetes depends on the allostatic response of pancreatic beta cells to synthesise and secrete enough insulin to compensate for insulin resistance. The endocrine pancreas is a plastic tissue able to expand or regress in response to the requirements imposed by physiological and pathophysiological states associated to insulin resistance such as pregnancy, obesity or ageing, but the mechanisms mediating beta cell mass expansion in these scenarios are not well defined. We have recently shown that ob/ob mice with genetic ablation of PPARγ2, a mouse model known as the POKO mouse failed to expand its beta cell mass. This phenotype contrasted with the appropriate expansion of the beta cell mass observed in their obese littermate ob/ob mice. Thus, comparison of these models islets particularly at early ages could provide some new insights on early PPARγ dependent transcriptional responses involved in the process of beta cell mass expansion

**Results:**

Here we have investigated PPARγ dependent transcriptional responses occurring during the early stages of beta cell adaptation to insulin resistance in wild type, ob/ob, PPARγ2 KO and POKO mice. We have identified genes known to regulate both the rate of proliferation and the survival signals of beta cells. Moreover we have also identified new pathways induced in ob/ob islets that remained unchanged in POKO islets, suggesting an important role for PPARγ in maintenance/activation of mechanisms essential for the continued function of the beta cell.

**Conclusions:**

Our data suggest that the expansion of beta cell mass observed in ob/ob islets is associated with the activation of an immune response that fails to occur in POKO islets. We have also indentified other PPARγ dependent differentially regulated pathways including cholesterol biosynthesis, apoptosis through TGF-β signaling and decreased oxidative phosphorylation.

## Background

Although the hallmark of obesity associated type 2 diabetes (T2D) is the decrease in insulin sensitivity, the development of hyperglycemia requires the failure of the allostatic response of the β-cells to respond by producing enough insulin to overcome the functional defect in insulin action [1]. One of the strategies the endocrine pancreas uses to adapt to changes in insulin resistant requirements associated with different physiological states, such as pregnancy, obesity, or ageing, is to expand the β-cell mass. Thus, in all these states insulin resistance leads to an increased production of insulin to maintain euglycemia [2]. Despite the increased requirements, the majority of individuals remain euglycemic by adequately increasing their β-cell mass and by adjusting their stimulated insulin secretion. However, when the allostatic β-cell adaptation fails, hyperglycemia will develop. Under conditions of allostatic overload, there is an association between pregnancy with gestational diabetes and obesity and ageing with T2D [3].

In humans and animal models, it has been widely recognised that β-cell failure is an essential factor leading to T2D. This can occur when β-cells fail to appropriately expand and/or to optimise their function, ultimately compromising in glucose-stimulated insulin secretion (GSIS). Animal models of insulin resistance are excellent models to demonstrate the plasticity of β-cell mass and provide suitable experimental systems in which to identify the extracellular signals and molecular mechanisms behind this compensatory response.

Valuable insights into the key role of β-cell failure in the pathogenesis of T2D has come from genome-wide association studies, an important resource to identify new unexpected susceptibility gene candidates for the development of T2D [4]. Of interest these studies identified validated variants associated with insulin-secretory defects in the general population and showed little if any relationship to insulin resistance [5-9].

Peroxisome proliferator-activated receptor gamma (PPARγ) is a member of the nuclear receptor superfamily of ligand-activated transcription factors [10] and has been shown to be involved in many diverse biological processes, including adipogenesis and glucose and lipid metabolism. In addition to these roles, it has been shown that PPARγ also exerts an important role in controlling cellular proliferation in different organs including pancreatic endocrine tissue. As already demonstrated, the absence of PPARγ specifically in β-cells cannot fully compensate for the β-cell dysfunction seen in states of peripheral insulin resistance [11]. In fact, animals with lack of PPARγ had blunted the physiological expansion of β-cell mass in response to high-fat feeding.

There are important differences between the mechanisms controlling the increase of β-cell mass observed during pregnancy and obesity suggesting certain degree of etiopathogenic specificity on the mechanisms controlling β-cell mass expansion [12-15]. We have recently shown that β-cell mass adaptation to insulin resistance failed in the adult POKO mice, an insulin resistant mouse resulting from the deletion of PPARγ2 in an obese ob/ob background [16]. This impaired β-cell mass expansion contrasted with the massive expansion observed in their obese littermate ob/ob mice. Thus, we rationalised that comparison of ob/ob and POKO mice islets particularly at early ages could provide some new insights on early PPARγ dependent transcriptional responses involved in the process of β-cell mass expansion.

To date, the main approach to identifying global transcriptional changes has been the use of gene expression microarray platforms in advanced stages of the disease. Here we focused in early evolutive stages to increase our chances of identifying novel early events and their molecular effectors involved in the adaptation of β-cells to insulin resistance. Our strategy has been to isolate total RNA samples from islets of 5-week-old female wild type (WT), PPARγ2KO, ob/ob and POKO mice for large-scale expression profiling. We have used 5-week-old mice to elucidate pathways and factors underlying the early islet proliferative response that might have failed in the POKO mice and that could cause the inadequate β-cell expansion in this model. Although at this age metabolic disturbances have not been identified in islets yet, insulin resistance is already detectable in ob/ob and POKO mice. In support of this approach we have shown that defects in proliferation markers can be detected at 5 weeks of age in POKO islets, well before alterations in the insulin secretory capacity were evident [17].

Initially, we have analyzed genes to compare ob/ob with WT mice. This comparison should identify any gene regulation in ob/ob mice that may be linked to the increased expansion of their β-cells. Secondly, we have compared POKO vs. WT islets. As these two models have a similar degree of expansion of their β-cells, the aim was to identify default genes induced during insulin resistance that were dependent on PPARγ2 activity. In our previous study [17] we have shown that at 16 weeks of age, despite being more insulin resistant than ob/ob mice, the β-cell mass of POKO mice remained similar to that of WT islets. This resulted in POKO mice having an inappropriately lower insulin plasma level than the ob/ob controls at 16 weeks of age [18]. The final comparison that we have analyzed was between POKO and ob/ob islets, representing different states of insulin resistance that could identify PPARγ-dependent genes involved in expansion and function of β-cell.

In this study we have identified genes that are downregulated in PPARγ-deficient POKO islets that are related to the proliferation and survival mechanisms that may facilitate the expansion of β-cell mass under insulin resistant conditions. We have also identified new pathways that may contribute to this adaptation such as immune response, cholesterol biosynthesis, apoptosis through TGF-β signaling pathway and decreased oxidative phosphorylation. Failure of these adaptive mechanisms may contribute to failure of β-cell function in POKO islets and may be dependent on PPARγ expression and functionality.

## Results

**Figure 1:**
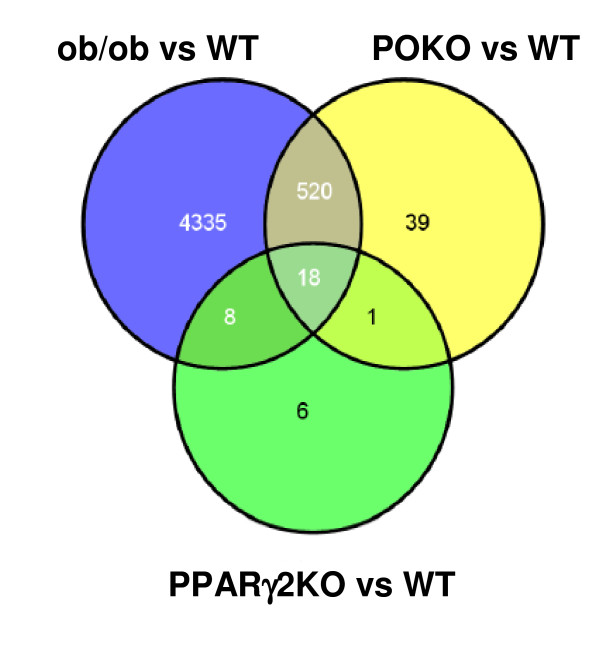
**A venn diagram showing relations between differentially expressed genes**. A venn diagram showing relations between differentially expressed genes (logFC ≥ 1; Adj. P-value ≤0.05) in the comparisons ob/ob vs. WT, POKO vs. WT and PPARγ2KO vs. WT mice.

Total RNA was extracted from islets obtained from 5-week-old female WT, PPARγ2KO, ob/ob and POKO mice and used for microarray analysis. The number of genes differentially expressed amongst the specific comparisons for an adjusted p-value ≤0.05, (unless otherwise specified), are shown in table 1 and the interactions between the diverse comparisons are shown in Figure 1.

**Table 1:** Differential expressed genes amongst the specific comparisons

	ob/ob vs. WT	PPARγ2KO vs WT	POKO vs. WT	ob/ob vs. PPARγ2KO	POKO vs. ob/ob	POKO vs. PPARγ2KO
**Down**	2349	7 (p ≤ 0.2)	51	23	1 (p ≤ 0.3)	81 (p ≤ 0.2)
**Up**	2532	26 (p ≤ 0.2)	527	30	3 (p ≤ 0.3)	85 (p ≤ 0.2)

Number of differentially expressed genes amongst the specific comparisons for an adjusted p-value ≤0.05 and log FC ≥ 1, unless are specified.

### Differential gene expression between wild type and ob /ob mice islets

We have previously shown that ob/ob mice have increased number and size of islets compared with their WT littermates [17,18], a difference that becomes more obvious as these mice age and become more metabolically compromised. Based on these observations, we hypothesised that the comparison between WT and ob/ob islets at a young age may identify early initial changes in islet gene expression associated with or leading to β-cell mass expansion (Table 2).

**Table 2:** Differential gene expression between WT and ob/ob islets and between WT and POKO islets

Comparison WT vs. ob/ob				
Name	Symbol	Description	Log FC	Adj p-value
NM_009828	Ccna2	cyclin A2 (Ccna2), mRNA	1.062995	0.037082104
NM_172301	Ccnb1	cyclin B1 (Ccnb1), mRNA	1.093047367	0.033661413
S78355	Ccnd1	Cyl-1 = cyclin D1 [mice, BALB/c, brain, mRNA, 3737 nt].	0.814358	0.060937249
NM_007632	Ccnd3	cyclin D3 (Ccnd3), transcript variant 1, mRNA	0.537596	0.081161755
NM_178674	Fbxl21	F-box and leucine-rich repeat protein 21 (Fbxl21), mRNA	1.379136	0.021465994
NM_207238	Fbxo27	F-box protein 27 (Fbxo27), mRNA	1.251021	0.024989279
NM_010583	Itk	Mus musculus IL2-inducible T-cellkinase (Itk), mRNA	4.9433924	0.016907267
NM_008764	Tnfrsf11b	tumor necrosis factor receptor superfamily, member 11b (osteoprotegerin) (Tnfrsf11b), mRNA	2.137707	0.00427033
NM_009689	Birc5	baculoviral IAP repeat-containing 5 (Birc5), transcript variant 1, mRNA	0.749711	0.0488386
NM_015793	Fbxw14	F-box and WD-40 domain protein 14 (Fbxw14), mRNA [NM_015793]	2.053845	0.020165243
NM_009873	Cdk6	cyclin-dependent kinase 6 (Cdk6), mRNA	-1.44108	0.011012
AK145759	Cdk7	blastocyst cDNA, RIKEN full-length enriched library, clone:I1C0038J11 product:cyclin-dependent kinase 7 (homolog of Xenopus MO15 cdk-activating kinase), full insert sequence	-1.18402	0.009851
**Comparison WT vs. POKO**				
**Name**	**Symbol**	**Description**	**Log FC**	**Adj p-value**

NM_008764	Tnfrsf11b	tumor necrosis factor receptor superfamily,member 11b (osteoprotegerin)(Tnfrsf11b), mRNA	1.4263697	0.028955326
X82786	mKi67	mRNA for Ki-67.	1.5311213	0.026624778
NM_009160	Sftpd	surfactant associated protein D (Sftpd), mRNA	2.10936363	0.04279018
NM_146187	Ffar2	free fatty acid receptor 2 (Ffar2), mRNA	1.371649	0.033601951

As expected the comparison between islets from ob/ob and WT mice identified many of the genes previously identified that play a role in β-cell expansion during states of insulin resistance such as obesity and pregnancy [15]. These positive controls included genes involved in proliferation, cell cycle and survival mechanisms [19]. For instance, gene expression levels of cyclin A2, B1, D1 and D3 were significantly increased in islets from ob/ob compared with WT mice. Conversely expression of cell division protein kinase 6 (Cdk6) and Cdk7 were significantly decreased in the ob/ob compared to WT islets. This transcriptional pattern closely mirrors the pattern observed during the β-cell hypertrophy typically associated with pregnancy, suggesting these genes are part of a common proliferative response associated with modulation of β-cell mass in physiological and pathophysiological states. Our analysis also confirmed the upregulation of genes encoding for enzymes involved in regulation of β-cell expansion (IL2-inducible T-cell kinase (Itk), Tumor necrosis factor receptor superfamily, member 11b (osteoprotegerin or Tnfrsf11b), baculoviral IAP repeat containing 5 (Birc5)) and cell cycle regulation (F-box and leucine-rich repeat protein 14 (Fbxw14), Fbxl21 and F-box protein 27 (Fbxo27)). Globally, these data indicate that at this early age the ob/ob islets have already activated their proliferative machinery and this data can be considered as a positive quality control for the experiment.

### Differential gene expression between WT and POKO islets

We next compared the gene expression profile between POKO and WT islets (Table 2). Since the POKO mouse fails to expand its β-cell mass despite insulin resistance, we hypothesized that this list may reveal by default genes induced during insulin resistance that were dependent on PPARγ2 activity. Our first observation was that POKO and WT islets had a very similar gene expression profile. In fact the expression of many of the genes previously shown to be dysregulated in ob/ob islets were not significantly different in the analysis comparing POKO and WT islets. This is consistent with the maintenance of "normal" β-cell mass in POKO mice that fail to expand. However, through more detailed analysis, it revealed that despite the inability to expand their β-cell mass, POKO islets had induction of some of recognized mediators of β-cell expansion, as observed during both pregnancy and obesity. These included Tnfrsf11b, mKi67, Surfactant-associated protein D (Sftpd) and Free fatty acid receptor 2 (Ffar2). These genes were increased in both the comparison between ob/ob vs. WT, and also to a lesser extent in the comparison between POKO vs. WT. These data suggested a dysregulation of the homeostatic control of β-cell mass in POKO islets, and that the ability of POKO mice to appropriately induce an appropriate response to a metabolic stress is impaired, with no increased proliferation and activation of survival mechanisms in their islets. Globally considered, this indicates a partial response of PPARγ-deficient ob/ob islets to insulin resistance.

### Differential gene expression between ob/ob and POKO mice

We hypothesized that the POKO vs. ob/ob comparison could identify PPARγ2 islet target genes that were dysregulated during the early stages of insulin resistant states contributing to a lack of β-cell mass expansion. We have previously shown that at 16 weeks of age POKO mice have significantly decreased islet number and mass compared to ob/ob mice and more interestingly, the number and size of islets in POKO mice resembled the number and size of the islets of a wild type 16-week-old mouse [17,18]. Analysis of gene expression by RT-PCR showed that at 5 weeks of age, there were already some changes in the expression of specific genes relating to proliferation (CyclinD1) and β-cell function (Insulin, MafA, PDX1 and Glut 2), with no evident differences in insulin secretory defects between POKO and ob/ob [17]. However, differences in gene expression of other genes, which have been shown at 16 weeks were inconsistent at this age. Surprisingly the comparison between 5-week-old ob/ob and POKO islets on microarray data only identified 3 upregulated genes, (Actin alpha1 (Acta1), Thrombospondin 4 (Thbs4) and Solute carrier family 5 (sodium/glucose cotransporter) (Slc5a10), and as expected, down regulation of gene PPARγ with an adjusted p-value ≤0.3 (Table 1). To allow a more detailed comparison between our models, we performed pathway analysis using Fatigo and FatiScan tools, enabling significant multiple comparisons of genes in significant pathways despite not having differentially specific expressed genes from these pathways at this early age.

### Dysregulated gene expression pathway analysis in models with different degree of beta cell expansion

A subsequent post-analysis was performed on the microarray data from the comparison between 5-week-old islets in order to identify the main dysregulated pathways, relating to the expansion of β-cells between the three genotypes. For this we have used two bioinformatic tools: the Ingenuity Pathway Analysis (IPA) and gene enrichment test from Babelomics tools (Fatigo), using an adjusted p value ≤0.05. To allow reliable interpretation of our data we have made the following comparisons: ob/ob vs. WT; associated with the normal adaptation to obesity induced insulin resistance with an expansion of the β-cell; and POKO vs. WT; associated with a failure in the adaptive response, with no expansion of β-cell. Using these two lists, we analyzed up-regulated and down-regulated genes from the comparison between ob/ob and WT, which were not regulated between POKO and WT.

First we analyzed the list of significantly up-regulated genes in islets from the comparison between ob/ob and WT mice that were not significantly changed in the comparison between POKO and WT mice. This was hypothesised to identify genes that were up regulated during β-cell expansion during obesity in the case of the ob/ob, which failed to occur in a PPARγ-dependent model such as the POKO mouse. Up regulated functions scored are based on the number of Network Eligible molecules they contain the top associated disease and disorders networks of up regulated genes from IPA for this first comparison were: immunological disease, inflammatory disease and inflammatory response. Other top networks scored for this comparison are presented in table 3. Cellular Development, and Cellular Function and Maintenance were identified as the first top molecular and cellular function networks. Cell morphology, proliferation and development were also in this first comparison. Similarly, the top five canonical pathways that they were significant up-regulated included Autoimmune Thyroid Disease Signalling, Allograft Rejection Signalling and more importantly Type 1 Diabetes Mellitus Signalling (Table 3).

**Table 3:** Functions and canonical pathways as differentially expressed up-regulated genes in ob/ob vs. WT, with no significant changes in POKO vs. WT mice

Top Bio Functions		
Name	p-value	# Molecules
**Diseases and Disorders**		
Immunological Disease	7.66E-18 - 3.63E-03	250
Inflammatory Disease	5.95E-15 - 3.60E-03	263
InflammatoryResponse	2.28E-14 - 3.63E-03	144
Skeletal and Muscular Disorders	4.99E-12 - 3.63E-03	255
Connective Tissue Disorders	7.78E-12 - 2.44E-03	175
**Molecular and Cellular Functions**		
Cellular Development	1.45E-17 - 4.37E-03	197
Cellular Function and Maintenance	2.21E-15 - 3.58E-03	103
Cell-To-Cell Signaling and Interaction	9.41E-15 - 4.37E-03	202
Cellular Growth and Proliferation	3.93E-13 - 3.11E-03	173
Cellular Movement	1.94E-09 - 3.78E-03	143
**Physiological System Development and Function**		
Hematological System Development and Function	3.63E-16 - 4.04E-03	189
Hematopoiesis	3.63E-16 - 3.27E-03	112
Tissue Morphology	2.59E-15 - 3.60E-03	124
Cell-mediated Immune Response	1.22E-14 - 3.78E-03	92
Immune Cell Trafficking	2.25E-13 - 3.78E-03	118
**Top Canonical Pathways**		
**Name**	**p-value**	**Ratio**

Autoimmune Thyroid Disease Signaling	3.06E-13	17/61 (0.279)
Allograft Rejection Signaling	1.49E-12	16/59 (0.271)
Type I Diabetes Mellitus Signaling	4.79E-12	27/119 (0.227)
Graft-versus-Host Disease Signaling	3.57E-10	15/49 (0.306)
B Cell Development	8.43E-10	12/37 (0.324)

Functions and canonical pathways identified by IPA as differentially expressed up regulated genes in islets from ob/ob vs. WT mice (fold change ≥ 1; P ≤ 0.05), with no significant changes in islets from POKO vs. WT mice.

Note: p value was calculated using the Benjamini-Hochberg method of multiple testing correction. In the case of canonical pathways "Ratio" indicates the number of differentially expressed genes in a given pathway, divided by total number of genes that make up that pathway.

**Figure 2:**
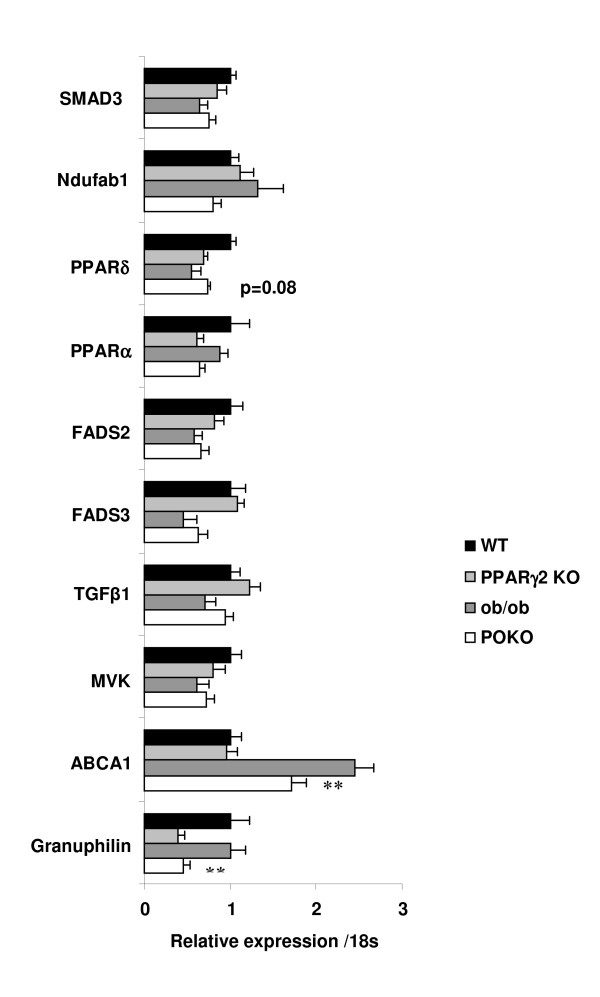
**Real time qRT-PCR results form genes to validate microarrays data**. Islet gene expression from 5-week-old female WT, ob/ob, PPARγ2KO and POKO mice (n = 8-11 mice per genotype). * p < 0.05 POKO vs. ob/ob.

Overall these results demonstrate that β-cell mass expansion in the context of obesity brings about a subtle, but coordinated increase in the basal level of a wide array of genes involved in the inflammatory and autoimmune response of ob/ob islets that does not occur in PPARγ2-ablated POKO islets. This concept was supported by the identification of specific molecules relating to antigen processing and antigen-presenting signalling such as MHC class II molecules and CD80/86 that were increased in the ob/ob vs. WT comparison and not the POKO vs. WT. Our analysis has also identified genes in these pathways such as granzyme B and perforin 1, with newly recognized roles in inflammation that have also been implicated in antigen presentation to cells, and genes such as granulophilin, which is involved in insulin secretion. Using databases of genome-wide regulatory module and element predictions (cisRED), we have identified a PPAR conserved sequence motif in the perforin 1 sequence and by validating decreased expression of granulophilin by RT-PCR in PPARγ2-ablated mice (Figure 2), it suggests a possible PPARγ dependent pathway.

We have also analysed genes that were down regulated in ob/ob islets when compared to WT, that were not significantly changed in the comparison between POKO and WT mice (Table 4). Genetic and Developmental Disorder, Dermatological and Gastrointestinal Disease were scored as top associated networks in this comparison, but organ development, growth and proliferation were also included in these networks. Significantly down regulated functions in this comparison were Lipid Metabolism, Small Molecule Biochemistry and Vitamin and Mineral Metabolism (Table 4). Expression of Tocopherol (alpha) transfer protein (TTPA), member RAS oncogene family (RAB27B), Acyl-CoA thioesterase 5 (ACOT5) and Hydroxysteroid (17-beta) dehydrogenase 14 (HSD17B14) were the most downregulated in ob/ob vs. WT, without changes between POKO vs. WT. Interestingly, we also identified a subset of genes from the canonical pathway of sterol biosynthesis including Mevalonate (diphospho) decarboxylase (MVD), Farnesyl diphosphate synthase (FDPS), Prenyldiphosphate synthase, subunit 1 (PDSS1), Isopentenyl-diphosphate delta isomerase 1 (IDI1), Cytochrome c oxidase 10 (COX10), Mevalonate kinase (MVK) and Fatty acid desaturase 3 (FADS3) to be significantly downregulated in ob/ob vs. WT, without changing in the comparison between POKO vs. WT (Table 4 and Figure 2).

**Table 4:** Functions and canonical pathways as differentially expressed down-regulated genes in ob/ob vs. WT, with no significant changes in POKO vs. WT mice

Top Bio Functions		
Name	p-value	# Molecules
**Diseases and Disorders**		
Dermatological Diseases and Conditions	3.01E-05 - 4.21E-02	37
Genetic Disorder	3.01E-05 - 4.21E-02	91
Cancer	9.80E-05 - 4.21E-02	170
Developmental Disorder	6.95E-04 - 4.21E-02	11
Gastrointestinal Disease	1.48E-03 - 4.21E-02	71
**Molecular and Cellular Functions**		
Lipid Metabolism	4.46E-07 - 4.21E-02	50
Small Molecule Biochemistry	4.46E-07 - 4.21E-02	71
Vitamin and Mineral Metabolism	4.46E-07 - 4.21E-02	23
Cell-To-Cell Signaling and Interaction	1.48E-04 - 4.21E-02	63
Cellular Assembly and Organization	1.48E-04 - 4.21E-02	46
**Physiological System Development and Function**		
Tumor Morphology	9.80E-05 - 4.21E-02	22
Hair and Skin Development and Function	1.37E-03 - 4.21E-02	26
Organ Development	1.37E-03 - 4.18E-02	35
Digestive System Development and Function	1.50E-03 - 1.95E-02	11
Organismal Development	1.50E-03 - 4.18E-02	26
**Top Canonical Pathways**		
**Name**	**p-value**	**Ratio**

Biosynthesis of Steroids	1.96E-04	7/128(0.055)
HER-2 Signaling in Breast Cancer	3.41E-03	9/79 (0.114)
Tight Junction Signaling	9.59E-03	13/167 (0.078)
Fatty Acid Biosynthesis	1.23E-02	3/51 (0.059)
ERK5 Signaling	1.26E-02	7/71 (0.099)

Functions and canonical pathways identified by IPA as differentially expressed down regulated genes in islets from ob/ob vs. WT mice (fold change ≥ 1; P ≤ 0.05), with no significant changes in islets from POKO vs. WT mice.

Note: p-value was calculated using the Benjamini-Hochberg method of multiple testing correction. In the case of canonical pathways "Ratio" indicates the number of differentially expressed genes in a given pathway, divided by total number of genes that make up that pathway.

We performed a gene enrichment test from Babelomics tools (Fatigo) on our data where significantly up and down-regulated groups of functionally related genes were identified from all expressed genes according to differential expression between the two comparisons (with higher or lower expression in ob/ob vs. WT but without changes in POKO vs. WT mice). Among the 1381 up-regulated genes in ob/ob vs. WT we identified 6 significantly (Adj. p-value ≤0.05) overrepresented GO terms, 11 InterPro and 9 KEGG pathways. Among the 957 down-regulated genes we identified 11 significantly (Adj. p-value ≤0.05) overrepresented GO terms and 3 KEGG pathways. Subsets of the functional groups are shown in Table 5, 6 and 7 and were chosen to include all significant (Adj. p-value ≤0.05) KEGG pathways, InterPro and all significant overrepresented functional groups satisfying GO. Confirming our initial data from IPA, there was an up-regulation of GO terms involved in the immune response and antigen binding such as the KEGG pathway positive regulation of immune response in ob/ob vs. WT, without any change in POKO vs. WT islets. The down-regulated functional groups in ob/ob vs. WT without any change in POKO vs. WT islets were mainly associated with structural genes and cell-junctions (See Additional file 1 Figure S1). Consistent with our previous data, we identified down-regulated cholesterol and sterol biosynthetic process and glutamine transport in the overrepresented GO terms in the comparison ob/ob vs. WT, without any significant difference in POKO vs. WT mice (Table 8 and Additional file 1 Figure S2).

**Table 5:** KEGG significant terms up-regulated in ob/ob vs. WT, without significant changes in POKO vs. WT mice

Term	Name	Term size	Odds ratio log	Adj. p-value
mmu00604	Glycosphingolipid biosybthesis	21	1.86	2.43E-02
mmu04940	Type 1 diabetes	54	1.83	2.96E-05
mmu05330	Allograt rejection	48	1.79	1.24E-04
mmu05332	Graft-verus host disease	50	1.74	1.63E-04
mmu05320	Autoinmune thyroid disease	63	1.71	3.08E-05
mmu04514	Cell adhesion molecules	141	1.65	2.05E-10
mmu05416	Viral myocarditis	80	1.23	5.78E-03
mmu04730	Long term depresion	74	1.14	2.51E-02
mmu04270	Vascular smooth muscle contact	126	1.12	1.43E-03

KEGG significant terms up-regulated in islets from ob/ob vs. WT mice (adjusted p-value ≤ 0.05), without significant changes in islets from POKO vs. WT mice.

**Table 6:** InterPro significant terms up-regulated in ob/ob vs. WT, without significant changes in POKO vs. WT mice

Term	Name	Term size	Odds ratio log	Adj. pvalue
IPR006116	2-5-oligoadenylate synthetase, ubiquitin-like region (IPR006116)	14	2.49	1.88E-02
IPR000342	Regulator of G protein signalling (IPR000342)	36	1.68	3.76E-02
IPR003597	Immunoglobulin C1-set (IPR003597)	93	1.62	5.28E-06
IPR006907	Protein of unknown function DUF622 (IPR006907)	73	1.51	1.05E-03
IPR003596	Immunoglobulin V-set, subgroup (IPR003596)	276	1.24	8.33E-09
IPR013151	Immunoglobulin (IPR013151)	364	1.22	3.35E-11
IPR003006	Immunoglobulin/major histocompatibility complex, conserved site (IPR003006)	90	1.17	4.29E-02
IPR007110	Immunoglobulin-like (IPR007110)	488	1.17	3.81E-13
IPR013106	Immunoglobulin V-set (IPR013106)	330	1.15	1.25E-08
IPR003599	Immunoglobulin subtype (IPR003599)	441	1.01	2.36E-08
IPR003598	Immunoglobulin subtype 2 (IPR003598)	371	0.89	7.22E-05

InterPro significant terms up-regulated in islets from ob/ob vs. WT mice (adjusted p-value ≤ 0.05), without significant changes in islets from POKO vs. WT mice.

**Table 7:** GO significant terms up-regulated in ob/ob vs. WT, without significant changes in POKO vs. WT mice

Name	Term size	Odds ratio log	Adj. pvalue
**GO molecular function**			
antigen binding (GO:0003823)	23	2.87	1.136E-06
**GO cellular component**			
cell surface (GO:0009986)	365	0.91	1.48E-07
external side of plasma membrane (GO:0009897)	190	1.41	2.38E-11
guanylate cyclase complex, soluble (GO:0008074)	5	1.8	6.95E-07
**GO biological process**			
immune response (GO:0006955)	599	0.83	7.288E-06
peptide hormone processing (GO:0016486)	13	2.93	4.08E-003

GO significant terms up-regulated in islets from ob/ob vs. WT mice (adjusted p-value ≤ 0.05), without significant changes in islets from POKO vs. WT mice.

**Table 8:** GO significant terms down-regulated in ob/ob vs. WT, without significant changes in POKO vs. WT mice

Name	Term size	Odds ratio log	Adj. pvalue
**GO molecular function**			
L-glutamine transmembrane transporter activity (GO:0015186)	5	4.55	1.27E-002
**GO cellular component**			
desmosome (GO:0030057)	24	2.47	2.39E-004
apical junction complex (GO:0043296)	132	1.82	1.17E-009
apicolateral plasma membrane (GO:0016327)	134	1.8	1.17E-009
tight junction (GO:0005923)	102	1.7	3.21E-006
cell-cell junction (GO:0005911)	219	1.4	2.54E-007
cell junction (GO:0030054)	540	0.88	1.57E-005
**GO biological process**			
cholesterol biosynthetic process (GO:0006695)	30	2.47	4.76E-004
sterol biosynthetic process (GO:0016126)	37	2.17	2.13E-003
tongue development (GO:0043586)	12	3.16	3.09E-003
glutamine transport (GO:0006868)	5	4.55	9,11E-003

GO significant terms down-regulated in islets from ob/ob vs. WT mice (adjusted p-value ≤ 0.05), without significant changes in islets from POKO vs. WT mice.

### Functional profiling of differentially expressed genes in POKO mice that may contribute towards failure to expand beta cell mass

We performed FatiScan analysis in the comparison between POKO and ob/ob islets obtained from our microarray. This FatiScan analysis highlights the value of using functional analysis, which incorporates all experimental data rather than limiting interpretation to those genes that rank among the highly differentially expressed. Using the FatiScan tool, 616 gene ontology groups, 85 InterPro, 67 KEGG pathways, 7 Biocarta, and 3 JasparTBS were observed to be significantly (adj. p value ≤ 0.05) overrepresented in islets from POKO relative to the ob/ob mice. There was a substantial overlap of genes within these identified functional groups resulting in the overrepresentation of a large number of functionally similar gene ontology groups. Therefore only the highly expressed groups have been shown in tables.

Initially, an overrepresentation of genes related to biological processes such as neutral lipid catabolic process, lipid glycosilation, collagen biosynthesis process and regulation of actin filament length were identified. Analysis from FatiScan also identified genes related to mitochondrial outer and inner membrane translocase complex and fibrillar collagen in the cellular component group and profilin, lipoic acid and IgG binding in the molecular function group. Other molecular functions associated with highly expressed genes in POKO islets compared with ob/ob were RPTP-like protein binding, platelet-derived growth factor binding and nucleocytoplasmic transporter activity. Genes from terms associated to Chaperonin TCP-1, Mob/phocein, hexokinase were identified as domains in InterPro.

**Figure 3:**
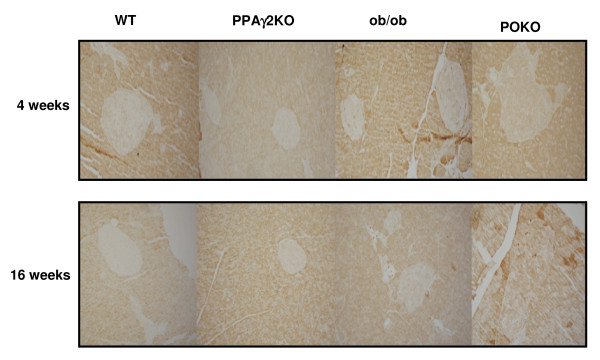
**Representative photomicrographs for TGF-ß immunostaining in pancreas sections at different age**. TGF-β immunostaining in pancreas sections from WT, ob/ob, PPARγ2KO and POKO mice at 4 and 16 weeks (n = 3-4 mice per genotype).

The most significant KEGG pathways identified in the FatiScan functional analysis of genes with higher expression in POKO compared to ob/ob islets were the olfactory transduction and spliceosome (Table 9). Although no significant differences in singular genes were detected between both genotypes, this analysis found that there was increased expression of pathways with genes related to the formation of cellular junctions between cells as regulation of actin cytoskeleton, tight, adherence and gap junction. Pathways identified with increased gene expression in POKO compared to ob/ob islets also included terpenoid backbone, glutathione metabolism, TGF-β signalling and steroid biosynthesis. Closer inspection of TGF-β1 and SMAD3 in 5-week-old POKO mice by RT-PCR surprisingly showed no significant increase in mRNA expression when compared to ob/ob mice (Figure 2). However we found increased significant immunostaining for TGF-β in POKO pancreas sections at 16 weeks of age, starting to be present at 4 weeks in POKO mice (Figure 3).

**Table 9:** KEGG significant terms up-regulated in POKO vs. ob/ob mice

ID	Term
mmu04740	Olfactory transduction
mmu03040	Spliceosome
mmu04810	Regulation of actin cytoskeleton
mmu04530	Tight junction
mmu04520	Adherens junction
mmu03050	Proteasome
mmu03010	Ribosome
mmu05219	Bladder cancer
mmu04512	ECM-receptor interaction
mmu05200	Pathways in cancer
mmu04670	Leukocyte transendothelial migration
mmu04115	p53 signaling pathway
mmu03020	RNA polymerase
mmu04020	Calcium signaling pathway
mmu00270	Cysteine and methionine metabolism
mmu00260	Glycine, serine and threonine metabolism
mmu00830	Retinol metabolism
mmu00240	Pyrimidine metabolism
mmu04510	Focal adhesion
mmu00900	Terpenoid backbone biosynthesis
mmu00100	Steroid biosynthesis
mmu00450	Selenocompound metabolism
mmu00480	Glutathione metabolism
mmu04012	ErbB signaling pathway
mmu04360	Axon guidance
mmu03420	Nucleotide excision repair
mmu04621	NOD-like receptor signaling pathway
mmu04144	Endocytosis
mmu04540	Gap junction
mmu00330	Arginine and proline metabolism
mmu04010	MAPK signaling pathway
mmu04062	Chemokine signaling pathway
mmu04110	Cell cycle
mmu00970	Aminoacyl-tRNA biosynthesis
mmu04350	TGF-beta signaling pathway
mmu00230	Purine metabolism
mmu03030	DNA replication
mmu00250	Alanine, aspartate and glutamate metabolism
mmu00561	Glycerolipid metabolism
mmu04060	Cytokine-cytokine receptor interaction
mmu00520	Amino sugar and nucleotide sugar metabolism
mmu00290	Valine, leucine and isoleucine biosynthesis
mmu00052	Galactose metabolism
mmu00040	Pentose and glucuronate interconversions
mmu03018	RNA degradation
mmu03320	PPAR signaling pathway

KEGG significant terms up-regulated in islets from POKO vs. ob/ob mice (adjusted p-value ≤ 0.05)

Pathways associated to genes with lower expression in POKO compared to ob/ob islets were also identified by Fatiscan (Table 10 Figure 2) such as those relating to oxidative phosphorylation, sulphur metabolism reduction and fixation, mature onset diabetes in the young, ATP-binding cassette (ABC) transporters and Type 1 and Type 2 diabetes. These results support the data obtained in our previous analysis between both the ob/ob vs. WT and POKO vs. WT comparisons. Furthermore, we have also verified significantly decreased expression of ABCA1 and an isoform of NADH dehydrogenase (Ndufab1) in the islets of 5-week-old POKO compared with ob/ob mice by RT-PCR (Figure 2).

**Table 10:** KEGG significant down-regulated in POKO vs. ob/ob mice

ID	Term
mmu00190	Oxidative phosphorylation
mmu05016	Huntington's disease
mmu05012	Parkinson's disease
mmu04142	Lysosome
mmu04270	Vascular smooth muscle contraction
mmu04730	Long-term depression
mmu04940	Type I diabetes mellitus
mmu02010	ABC transporters
mmu00510	N-Glycan biosynthesis
mmu04612	Antigen processing and presentation
mmu04720	Long-term potentiation
mmu05330	Allograft rejection
mmu04260	Cardiac muscle contraction
mmu04070	Phosphatidylinositol signaling system
mmu00920	Sulfur metabolism
mmu05320	Autoimmune thyroid disease
mmu04912	GnRH signaling pathway
mmu04930	Type II diabetes mellitus
mmu04914	Progesterone-mediated oocyte maturation
mmu04950	Maturity onset diabetes of the young

KEGG significant terms down-regulated in islets from POKO vs. ob/ob mice (adjusted p-value ≤ 0.05).

Carbohydrate responsive element-binding protein (Chrebp), casein kinase 1 (ck1) and insulin-like growth factor type 1 receptor (igfr1) pathways were the three highly expressed in Biocarta associated to genes with higher expression in POKO compared with ob/ob islets (data not shown).

FastiScan analysis identified 3 JasparTBS to be significantly (adj. p-value ≤ 0.05) overrepresented in islets from POKO relative to the ob/ob mice. The list of genes regulated by the transcription factors Pax2 and Arnt:Ahr were significantly expressed in POKO compared with ob/ob islets, while genes regulated by HOXA5 were down regulated and overrepresented in POKO islets (Table 11).

**Table 11:** Jaspar TFBS significant terms down-regulated in POKO vs. ob/ob mice

Term	Term size	Odds ratio log	Adj pvalue
Pax2	298	0.54	3.20E-02
Arnt::Ahr	456	0.47	3.20E-02
HOXA5	168	-0.74	2.88E-02

Jaspar Transcription Factor Binding Site (TFBS) significant terms down-regulated in islets from POKO vs. ob/ob mice (adjusted p-value ≤ 0.05).

### Dysregulation of genes dependent on PPAR in POKO and ob/ob islets

One of the pathways we found to be altered in FatiScan analysis between POKO vs. ob/ob was the PPAR pathway (Table 9). The expression of PPAR alpha and total PPAR gamma were decreased in the islets of POKO compared with ob/ob mice (Figure 2); however the expression of PPAR beta/delta were increased (Figure 2 and Additional file 1 Table S1). Furthermore, the expression of the three isoforms of the nuclear receptor Retinoic X receptor (RXR alpha, beta and gamma), heterodimers of PPARs, were differentially altered depending on the isoform as shown in the table. Most of the pathways analysed had increased or decreased expression of PPAR target genes as shown in Additional file 1 Table S1. However, gene expression of PPAR target genes involved in lipogenesis (SCD1, ME1, FADS2) and in cholesterol metabolism (CYP7A1, CYP8B1, LXRa and CYP27) were increased in POKO compared to ob/ob islets (Figure 2). Conversely, PPAR target genes involved in fatty acid oxidation (Ehhadh, Acaa1b, SCP2, Acox1, CPT1a, CPT1c and CPT2) were decreased suggesting these genes could be regulated by the PPARγ2 isoform, a pattern also observed with genes involved in adipocyte differentiation (Perilipin, aP2, adipoq, MMP-1) or gluconeogenesis (PEPCK, GyK, AQP7).

### Genome Wide candidate genes and early transcriptional response in pancreatic beta cells

Recent genome-wide association studies have provided an important resource for furthering our understanding of type 2 diabetes disease mechanisms [5,9]. These published variants were all associated with insulin-secretory defects in the general population and show little if any relationship to insulin resistance. We have checked these genes in both our comparisons (See additional file 1 Figure S3). Although expression of Slc30A8 was not altered in the two comparisons in our microarray data, we found that expression of Slc30A8 detected by RT-PCR was decreased in islets from POKO compared to ob/ob mice and in islets from PPARγ2KO compared to WT mice. Furthermore, using NSITE/Recognition of Regulatory motifs tool we found a PPAR conserved sequence motif in this gene suggesting this gene has a dependency on PPARγ.

We also checked expression of NR4A3 (also called Neuron-derived orphan receptor (Nor) 1) by RT-PCR in our models. This is a novel candidate gene for β-cell function, which was not covered by the SNP arrays of the recent genome-wide association studies for type 2 diabetes mellitus [20]. Here we found significantly increased expression of NR4A3 in ob/ob and POKO islets when compared with WT and PPARγ2KO islets, but no significant differences between the ob/ob and POKO islets (See Additional file 1 Figure S3).

Recent studies have shown that the Notch-regulated transcription factor neurogenin 3 (Ngn3) is critical for the development of endocrine cells of islets [21]. Ngn3 was increased in islets from ob/ob and POKO mice compared with those from PPARγ2KO and WT, but with no significant differences between the former genotypes. From the current GWAS and metaanalysis in Europeans associated to adiposity loci [22], we identified increased NEGR1 expression in our microarray data between ob/ob and WT islets and lower expression in POKO when compared to ob/ob islets after analysis by RT-PCR.

## Discussion

In this study we have used a gene expression microarray and a subsequent bioinformatic approach to identify new molecular mechanisms and pathways implicated in the expansion of pancreatic β-cells in the context of insulin resistance. Using this technique and bioinformatic analysis we have investigated: 1) the overrepresentation of genes related to β-cell expansion under situations of insulin resistance in islets, 2) the genes differentially expressed in insulin resistant islets with and without the capacity of β-cell to expand, and 3) the overrepresentation of functional groups of genes differentially expressed previously to normal o defective expansion of β-cell mass.

We first examined genes that were upregulated in the islets of ob/ob compared to WT mice, expecting to identify pathways relevant to β-cell mass expansion for future comparison with the POKO mouse which is unable to expand its islets. We found that, at the age of 5 weeks, the ob/ob islets already exhibit induction of several pathways relating to cell proliferation and survival mechanisms. This response was not observed in POKO islets suggesting that these regulatory mechanisms are activated very early on before severe metabolic stress has developed, and that a failure to initiate these pathways is effectively contributing to the failure of the homeostatic control of β-cell mass in POKO islets. Specifically, POKO islets did not show the same induction of cyclin A2, B1, D1 and D3 observed in ob/ob islets, where in addition to their role in cell cycle progression, new data has revealed an emerging role of D-type cyclins in transcriptional regulation and cellular differentiation processes. There is also evidence from studies in 3T3-L1 cells that cyclin D3 acts as a ligand-dependent PPARγ coactivator and that its expression is increased throughout the adipocyte differentiation process [23]. Our results could therefore suggest that regulation of cyclinD3 is at least partly dependent on PPARγ which may be of relevance in the proliferative mechanisms of β-cell mass. Other universal genes previously shown to be increased during the expansion of the β-cell during obesity or pregnancy such as Birc5, Igf1r and Rasgrp1 also failed to increase in PPARγ deficient ob/ob islets (POKO vs. WT) further suggesting an inability of POKO islets to proliferate at a young age.

We were interested in identifying a putative molecular mechanism that could induce the pancreatic islet dysfunction in POKO mice. Our experimental design allowed the identification of pathways associated with the normal adaptation of islets to obesity induced insulin resistance (comparison ob/ob vs. WT) and pathways associated with failure in this response (comparison POKO vs. WT). We have identified genes associated with canonical pathways of Autoimmune Thyroid Disease Signalling, Allograft Rejection Signalling and Type 1 Diabetes Mellitus Signalling are upregulated in ob/ob vs. WT islets. This was associated with a significant increase of genetic programmes related to inflammatory disease, inflammatory response and immunological disease. It is well established that inflammation and fibrosis are processes preceding the deterioration of pancreatic islet structure. Molecules from MHCII (HLA-D, HLA-A, HLA-B) and other molecules such as CD80/86, which are involved in the antigen presentation signalling were increased in ob/ob relative to WT islets, with no significant changes in the islets of POKO vs. WT, suggesting that their involvement in remodelling is linked to β-cell mass expansion. As these pathways are activated in ob/ob islets and not in POKO islets, and that this is occurring very early before ob/ob islets fail, we speculate that this immune and inflammatory response may in fact be a physiological response, initiating or enabling a mechanism of remodelling or adaptation in order for the β-cell mass to expand. Thus it is conceivable that these changes are part of an adaptive response required under metabolic stress as occurs in ob/ob mice, possibly involving signalling via PPARγ.

Our FatiScan analysis showed that TGF-β/Smad signaling pathway was significantly increased in POKO compared to ob/ob islets, which is supported by increased pancreatic staining of TGF-β in POKO islets at 4 and 16 weeks of age. It has already been shown in other organs such as kidney that stimulation of fibrotic processes could be mediated, at least in part, by a down-regulation of PPARγ that can favour an up-regulation of the TGF-β/Smad signalling pathway [24,25]. Genes such as TGF-β1, its receptors type I and II and SMAD2/3/4, also implicated in apoptosis and cell cycle arrest through this pathway were increased in POKO islets compared to ob/ob islets, suggesting that specific profibrotic and apoptotic genes could also be involved in the failure of β-cell expansion in POKO islets.

Two genes, perforin 1 and granzyme B which are required for the direct recognition of the cells [26] were also up regulated in our ob/ob vs. WT comparison. The dominant role of the perforin/granzyme pathway in β-cell destruction in type 1 diabetes and allogeneic islet graft rejection makes this pathway an important target for future therapies for type 1 diabetes. However our results and the suggested new role of these molecules in inflammation also suggests that they could be new targets for treatments of type 2 diabetes, as part of their role includes the adaptation of the β-cell to insulin resistance. Indeed they could contribute to the remodelling mechanisms enabling the necessary expansion of the pancreatic β-cell by controlling apoptosis in a PPARγ dependent pathway. Supporting this idea, we have found that the perforin promoter has a PPAR response element and that POKO islets lack this immune response, further supporting its role facilitating the remodelling adaptation in response to insulin resistance. Globally considered our data indicate that PPARγ-ablated islets (POKO) may have decreased capacity for remodelling and an exacerbated proapoptotic response ultimately leading to their failure.

Besides an increased β-cell mass during the adaptation to insulin resistance, the most immediate response is the optimisation of the insulin secretion. The data from this study showed that cholesterol-induced impairment of β-cell function could occur in POKO islets as happens in other models [27]. It has already been shown that Ldlr-/- knockout mice with β-cell-specific ATP-binding cassette transporter A1 (ABCA1) deficiency showed increased islet cholesterol content and β-cell dysfunction, suggesting that cholesterol efflux through ABCA1 is a critical regulator of islet cholesterol content and β-cell function [28]. Functional analysis using FatiScan and our RT-PCR results revealed increased expression of genes related to cholesterol and sterol biosynthesis and decreased expression of ATP-binding cassette (ABC) transporters in POKO compared with ob/ob islets. Also rosiglitazone, an activator of the peroxisome proliferator-activated receptor-gamma, requires β-cell ABAC1 for its beneficial effects on glucose tolerance. It has been suggested that elevated β-cell cholesterol levels may impair insulin secretion by promoting the dimerisation of neuronal NO synthase (nNOS), which downregulates glucokinase (GK), thus impairing glucose sensing. This will agree with our previous observation showing that GK is downregulated in POKO compared with ob/ob islets [17]. Moreover, it has been suggested that cholesterol also inhibits steps in insulin exocytosis and that ABCA1 could have other independent effects on insulin secretion. Here we show that granulophilin is downregulated in POKO islets and its expression depends on PPARγ similarly to GK. Thus we suggest that PPARγ could play a main role determining the cholesterol flux and its toxicity on the islet insulin secretion machinery under insulin resistance conditions.

Another pathway down regulated in POKO in comparison to ob/ob islets was oxidative phosphorylation. Different isoforms of Cytochrome c oxidase and NADH dehydrogenase were downregulated in POKO compared to ob/ob islets suggesting impaired complexes of the mitochondrial oxidative phosphorylation system in POKO islets could contribute to the failure of their islets. It has been shown recently in human islets and cell lines [29], that increased levels of nitrogen species may interact with the insulin exocitosis mechanism. From our previous data, we did not observe increased levels of ROS in POKO mice at this age; however nitrogen species under pathophysiological conditions such as chronic hyperglycemia could result in insulin secretion dysfunction in POKO islets.

Actin cytoskeleton remodelling is known to be involved in glucose-stimulated insulin secretion (GSIS). Cell-cell contacts mediated by intercellular junctions are crucial for proper insulin secretion in the endocrine pancreas. FatiScan analysis found that the tight junction, adherens junction and focal adhesion pathways were increased in POKO compared to ob/ob islets. In fact expression of Actin 1 alpha, which is involved in focal adhesion, was increased in POKO islets. Of note activation of actin cytoskeleton could result in increased fibrosis in the islets of POKO mice in a process facilitated by TGF-β/Smad activation as has been similarly shown in liver fibrosis [30].

Genome-wide association studies (GWAS) have had success in identifying loci that are involved in common diseases such us diabetes and interestingly some locus were linked specifically with T2D. Mouse models offer a good tool to help to take data from GWAS studies further investigating the function of associated genes *in vivo*. We further investigated some of these T2D susceptibility loci identified thorough GWAS in our models. We found only significant dowregulation in GK and SLC30A8 in POKO in comparison to ob/ob islets, both dependent on PPARγ expression. Specific β-cell inactivation of SLC30A8 in mouse results in glucose intolerance [31] and recently published meta-analysis results revealed a significant association between the rs13266634 C/T polymorphism of SLC30A8 and T2DM and IGT [32]. Our results suggest that SLC30A8 might therefore have a role in expansion of the β-cell and benefit from PPARγ agonists in the treatment of type 2 diabetes.

## Conclusions

Using transcriptome tools and mouse models of insulin resistance, in this study we show: a) that mechanisms of proliferation and survival signals were downregulated in POKO islets from an early age, b) we also identified new pathways that could be involved in the adaptation of the β-cell to insulin resistance during obesity and that they are defective in PPARγ-deficient ob/ob islets. Our results indicate that early stages of β-cell mass expansion are associated with activation of an inflammatory immune response that could facilitate the necessary remodeling while preventing activation of an apoptotic cascade. Other potential relevant pathogenic mechanisms modulating β-cell mass include cholesterol toxicity, TGF-β/Smad pathway-induced apoptosis/fibrosis and defective oxidative phosphorylation. As the pathways identified in our POKO islets are involved in the failure of β-cell function, our results indicate an important role of PPARγ in islets, modulating the adaptive response of β-cell mass to increased metabolic demands imposed by obesity and insulin resistance.

## Methods

### Animal care

Animals were housed in a temperature-controlled room (20-22 8C) with 12-h light/dark cycles. Food and water were available ad libitum unless noted. All animal protocols used in this study were approved by the UK Home Office and the University of Cambridge and by Ethical and Veterinary committees of Rey Juan Carlos University in Spain.

### Isolation and culture of pancreatic islets

The pancreas was injected, though the bile duct, with cold Hank's solution containing 0.4% (w/v) collagenase P (Roche Biochemicals). The pancreas was removed, digested for 8 minutes, and islets were collected by hand picking. Isolated islets were cultured overnight in cell medium (RPMI 1640 with 10% FBS, and 100 UI/ml penicillin, 100 μg/ml streptomycin), at 37°C, in 5% CO2 in air. For RNA extraction, islets were used on the day after isolation.

### RNA isolation and purification

An average of 25-50 islets were used per sample and total RNA was extracted according to the manufacturer's instructions. Each sample was purified using the RNeasy^® ^Mini kit (Qiagen Ltd, Crawley, UK). RNA was quantified using a NanoDrop^® ^ND1000 spectrophotometer V 3.5.2 (NanoDrop Technologies, Wilmington, DE). RNA quality was subsequently assessed using the 28S:18S ratio running the samples on a denaturing agarose gel. The 2:1 ratio (28S:18S) indicated that the RNA was intact.

### Sample labelling

One-Color Microarray-Based Gene Expression Analysis Protocol (Agilent Technologies, Palo Alto, CA, USA) was used to amplify and label RNA. A pool of 3-4 mice were used per each microarray and 3 microarrays were used per each genotype. Briefly, 400 ng of total RNA from WT, PPARγ2KO, ob/ob and POKO mice, were reverse transcribed using T7 promoter primer and the Moloney murine leukemia virus (MMLV) reverse transcriptase. cDNA was then converted to anti-sense RNA (aRNA) by using T7 RNA polymerase that amplifies target material and incorporates cyanine 3 (Cy3)-labeled CTP simultaneously.

### Sample hybridization and image analysis

Samples were hybridized to a Whole Mouse Genome Microarray 4x44K (G4122F, Agilent Technologies). 1.65 micrograms of Cy3-labeled aRNA were hybridized for 17 hours at 65°C in an Agilent hybridization oven (G2545A, Agilent Technologies) set to 10 rpm in a final concentration of 1x GEx Hybridization Buffer HI-RPM (Agilent Technologies). Arrays were washed and dried out using a centrifuge according to manufacturer's instructions (One-Color Microarray-Based Gene Expression Analysis, Agilent Technologies). Arrays were scanned at 5 mm resolution on an Agilent DNA Microarray Scanner (G2565BA, Agilent Technologies) using the default settings for 4x44k format one-color arrays. Images provided by the scanner were analyzed using Feature Extraction software v10.1.1.1 (Agilent Technologies).

### Quality control

Based on statistical outliers criteria implemented in the Bioconductor package "ArrayQualityMetrics" there were no statistical outliers in terms of MA plots, dendrogram or boxplots.

### Statistical analysis

For the normalization, Agilent Processed Signal (Agilent Feature Extraction Software) was standardized across arrays using quantile normalization [33].

Differential gene expression was carried out using the limma [34] package from Bioconductor. This approach fits a linear model to all data considering as fixed effect the type of mice and using the array as blocking variable. Limma calculates moderated t-statistics, adding to the error term some information on the variance of all genes, solving the typical microarray problem of small sample size (2 in one of the experimental groups). Multiple testing adjustment of p-values was done according to Benjamini and Hochberg methodology [35].

The microarrays data of this study have been deposited in the Gene Expression Omnibus database under accession number GSE33647.

### Ingenuity Pathway Analysis

Functional and Canonical Pathway analyses of specific gene datasets were generated through the use of Ingenuity Pathway Analysis (Ingenuity Systems^®^, http://www.ingenuity.com).

The Functional analysis identified functions and/or diseases that were most significant to the dataset. Genes from the dataset that were associated with biological functions and/or diseases in the Ingenuity knowledge base were considered for the analysis. B-H Multiple Testing Correction p-value test was used to calculate a p-value determining the probability that each biological function and/or disease assigned to the data set is due to chance alone.

Genes associated with a canonical pathway in the Ingenuity knowledge database were considered for the analysis. The significance of the association between the dataset and the canonical pathway was measured in two ways: 1) A ratio of the number of genes from the dataset that map to the pathway divided by the total number of molecules that exist in the canonical pathway is displayed. 2) B-H Multiple Testing Correction p-value test was used to calculate a p-value determining the probability that the association between the genes in the dataset and the canonical pathway is explained by chance alone. IPA identified significant networks, top functions and canonical pathways associated with the differentially expressed genes for each comparison analyzed.

### FatiGO and FatiScan analysis

Enrichment analysis and gene set analysis was carried out for the Gene Ontology terms, InterPro terms and for the KEGG Pathways using FatiGO [36] and FatiScan [37] integrated in Babelomics suite [38].

FatiGO is a procedure to extract several functional terms as Gene Ontology (GO) terms, InterPro annotation or KEGG Pathways that are significantly over-or under-represented in sets of genes within the context of a genome-scale experiment. This resource is used to detect relevant functional terms for a group of genes with respect to a set of genes of reference (typically the rest of genes). The terms are considered to be relevant by the application of a Fisher's exact test that considers the multiple-testing nature of the statistical contrast performed.

FatiScan is a gene set analysis that detects significantly up-or down-regulated blocks of functionally related genes in lists of genes ordered by differential expression. FatiScan can search blocks of genes that are functionally related by different criteria such as gene ontology terms, Kyoto Encyclopedia of Genes and Genomes pathways, and others. The core of the method proposed is based on an algorithm to test whether a set of genes, labeled with terms (biological information), contain significant enrichments on one or several of these terms with respect to another set of genes of reference. FatiScan uses a Fisher's exact test for 2 × 2 contingency tables for comparing two groups of genes and extracting a list of GO terms whose distribution among the groups is significantly different. Given that many GO terms are simultaneously tested, the results of the test are corrected for multiple testing to obtain an adjusted p-value. FatiScan returns adjusted p-values based on False Discovery Rate (FDR) method [39,40].

GO and InterPro annotation for the genes in the microarray where taken from Ensembl 56 release and KEGG Pathways from the KEGG web page.

### RNA preparation and real-time quantitative PCR for validation of the microarray

Selected cDNA samples (n = 8-11) were quantified by real time quantitative PCR (qRT-PCR). Total RNA was isolated from islets samples according to the manufacturer's instructions following same protocols used for microarray RNA samples. Complimentary DNA was generated from 500 ng of RNA using M-MLV reverse transcriptase and master mix (Promega) in a 20 μl reaction with 2.5 mM MgCl2, 1.25 mM dNTPs and 5 μg/ml random hexamers at 37°C for 1 hour. cDNA was diluted 75 fold and 5 μl of diluted cDNA was used in a 12 μl real time PCR reaction using TaqMan primers and probes or SYBR green reagent (Applied Biosystems) according to manufacturers instructions. Reactions were run in duplicate for each sample and quantified in the ABI Prism 7900 sequence detection system (Applied Biosystems). Data was normalised to 18s rRNA. Primer sequences are shown in Additional file 1 Table S2.

### Immunohistochemistry of TGF-beta

Fixed tissue sections (4 μm) were dehydrated by graded ethanol's and xylene, and then embedded in paraffin (2-3 sections per animal, 3-4 animal per group). The sections were deparaffinized and rehydrated. Sections were incubated with anti-TGF-β(SantaCruz Biotechnology, INC.). Sections were incubated with a biotinylated anti-IgG (Vector Laboratories) and incubated with the avidin-biotin-peroxidase complex (Vector Laboratories). 3, 3'-diaminobenzidine (DAB) substrate (Sigma-Aldrich^®^) was used as the chromogen. Some samples were incubated without primary antibody as negative controls. The stained sections were imaged with a light microscope Zeiss Standard 25.

## Competing interests

The authors declare that they have no competing interests.

## Authors' contributions

YV, MR, AVP and GMG designed the experiment. YV, CMG, AI, IV, MC and GMG participated in the collection of samples. YV, IV, MC performed the RNA extractions. YV and MC performed the real time qRT-PCR experiments. FGG, JD, SC and AD were responsible for the bioinformatics. FGG and SC performed functional analysis of the data. YV, MR, FGG and SC assisted with manuscript preparation. AVP and GMG wrote the paper. GMG coordinated and supervised the project. All authors read and approved the final manuscript.

## Pre-publication history

The pre-publication history for this paper can be accessed here:

http://www.biomedcentral.com/1755-8794/4/86/prepub

## Supplementary Material

Additional file 1 **Figure S1 - GO Cellular Component significant terms**. GO Cellular Component significant terms (adjusted p-value ≤ 0.05) down-regulated in ob/ob vs. WT, without significant changes in POKO vs. WT. **Figure S2 - GO Biological Process significant terms**. GO Biological Process significant terms (adjusted p-value ≤ 0.05) down-regulated in ob/ob vs. WT, without significant changes in POKO vs. WT. **Figure S3 - Real time qRT-PCR results form genes from GWAS studies to validate microarrays data**. Islet gene expression from 5-week-old female WT, ob/ob, PPARγ2KO and POKO mice (n = 8-11 mice per genotype). * p < 0.05 POKO vs. ob/ob. **Table S1 - PPAR signalling pathway in POKO vs. ob/ob mice**. Genes from PPAR signalling pathway (adjusted p-value ≤ 0.05) in islets from POKO vs. ob/ob mice. **Table S2 - Table of primers and probes sequences used in RT-PCR validation**. Sequences of primers and probes (Syber-Green and Taqman) used in RT-PCR validation. Click here for file
