# Supplementary material for: Early peroxisome proliferator-activated receptor gamma regulated genes involved in expansion of pancreatic beta cell mass
Source: BMC Med Genomics. 2011 Dec 30;4:86. doi: 10.1186/1755-8794-4-86 (PMC3315430; doi:10.1186/1755-8794-4-86)
Supplement: Additional file 1 — Figure S1 - GO Cellular Component significant terms. GO Cellular Component significant terms (adjusted p-value ≤ 0.05) down-regulated in ob/ob vs. WT, without significant changes in POKO vs. WT. Figure S2 - GO Biological Process significant terms. GO Biological Process significant terms (adjusted p-value ≤ 0.05) down-regulated in ob/ob vs. WT, without significant changes in POKO vs. WT. Figure S3 - Real time qRT-PCR results form genes from GWAS studies to validate microarrays data. Islet gene expression from 5-week-old female WT, ob/ob, PPARγ2KO and POKO mice (n = 8-11 mice per genotype). * p < 0.05 POKO vs. ob/ob. Table S1 - PPAR signalling pathway in POKO vs. ob/ob mice. Genes from PPAR signalling pathway (adjusted p-value ≤ 0.05) in islets from POKO vs. ob/ob mice. Table S2 - Table of primers and probes sequences used in RT-PCR validation. Sequences of primers and probes (Syber-Green and Taqman) used in RT-PCR validation. [file 1755-8794-4-86-S1.DOC]

**Figure S1**


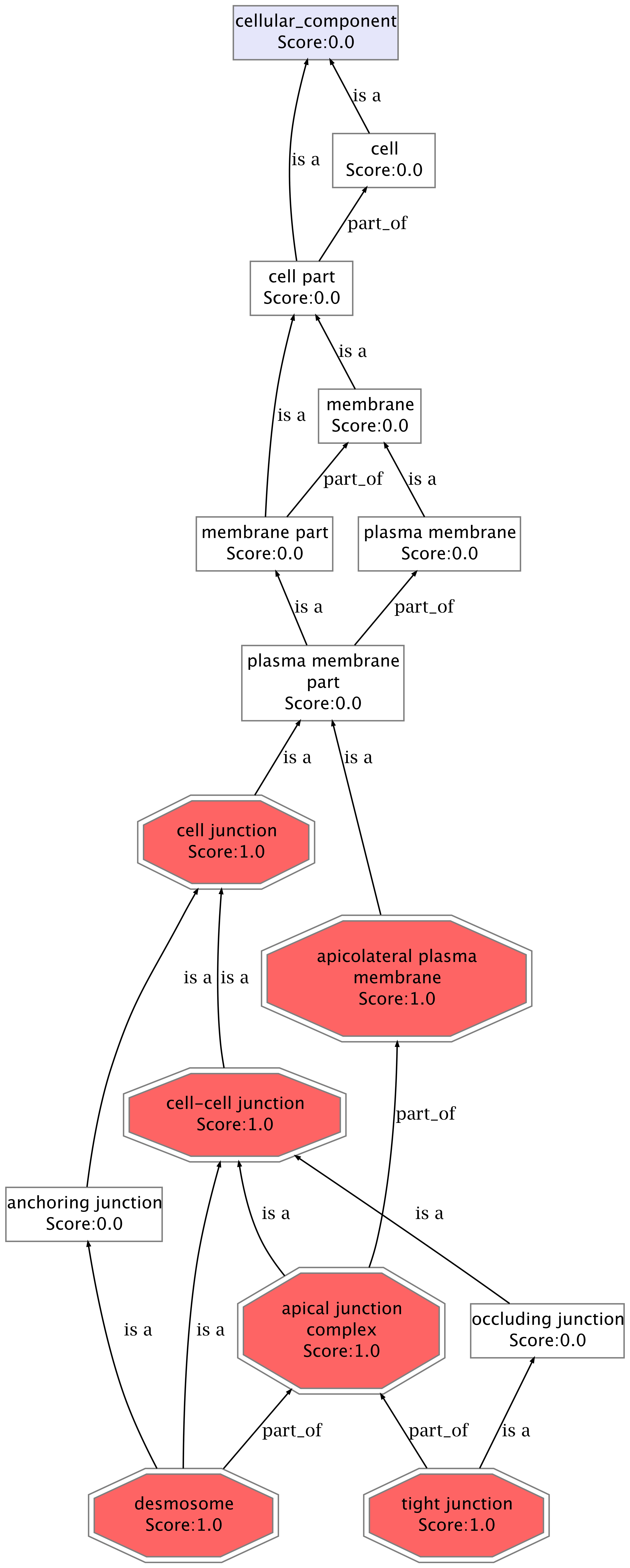


**Figure S2**


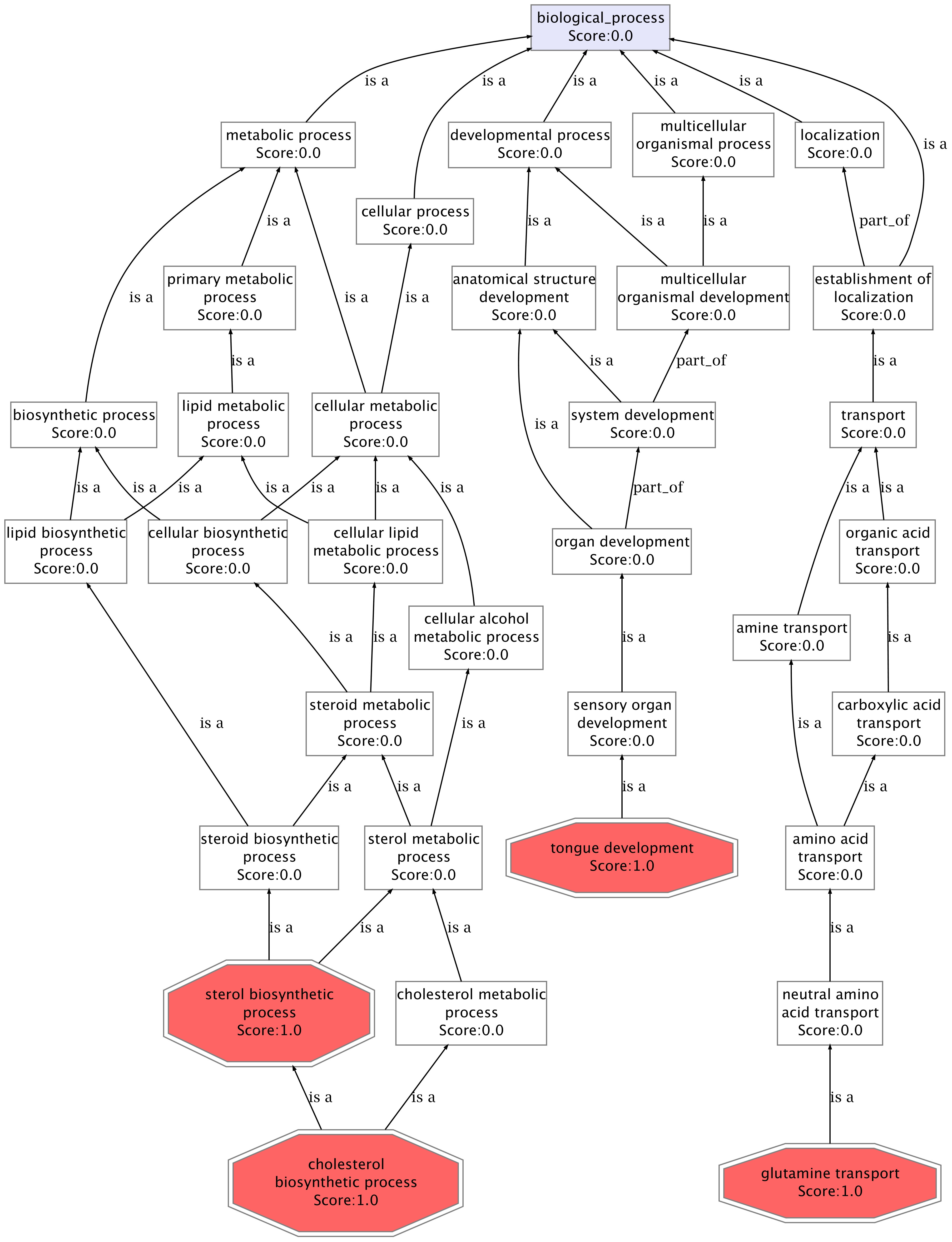


**Figure S3**

**0**

**0.5**

**1**

**1.5**

**2**

**2.5**

**3**

**NEGR1**

**IDE**

**NR4A1**

**NR4A2**

**NR4A3**

**SLC30A8**

**NOTCH2**

**NGN3**

**Relative expression /18s**

**WT**

**PPARγ2 KO**

**ob/ob**

**POKO**

*****

*****

*****

**Table S1 - PPAR signalling pathway in POKO vs. ob/ob mice**

| **gene** | **mgi_automatic_gene_symbol** | **t.statistic** | **p.value** | **p.adjusted** |
| --- | --- | --- | --- | --- |
| NM_001037999 | diazepam binding inhibitor (Dbi) | 2.81188242 | 0.01984197 | 0.56436654 |
| NM_053115 | Acox2 | 2.53188306 | 0.03155146 | 0.56436654 |
| NM_030721 | Acox3 | 2.27103984 | 0.04859111 | 0.56746944 |
| NM_010011 | cytochrome P450, family 4 | 2.12028472 | 0.06226674 | 0.58033181 |
| NM_017399 | Fabp1, liver | 1.95831568 | 0.08107695 | 0.60561396 |
| NM_007980 | Fabp2 | 1.89098739 | 0.09038833 | 0.62275451 |
| NM_009128 | Scd2 | 1.79080324 | 0.10611454 | 0.64571972 |
| AK165571 | Slc27a1 | 1.7785694 | 0.1082001 | 0.64747785 |
| X70100 | keratinocyte lipid-binding protein | 1.75230736 | 0.11280547 | 0.65627996 |
| NM_177406 | cytochrome P450, family 4, subfamily a, polypeptide 12a (Cyp4a12a) | 1.74845781 | 0.1134955 | 0.65666298 |
| NM_007382 | acyl-Coenzyme A dehydrogenase, medium chain | 1.54018073 | 0.15707832 | 0.70050976 |
| NM_011145 | peroxisome proliferator activator receptor delta | 1.53961551 | 0.15721462 | 0.70050976 |
| NM_207625 | acyl-CoA synthetase long-chain family member 4 (Acsl4) | 1.44863821 | 0.1805558 | 0.7032151 |
| NM_019699 | Fads2 | 1.40293833 | 0.19337829 | 0.70984545 |
| NM_009463 | Ucp1 | 1.36795233 | 0.20371577 | 0.71731026 |
| NM_009692 | apolipoprotein A-I (Apoa1), | 1.33797348 | 0.21294405 | 0.72338785 |
| NM_011989 | solute carrier family 27 (fatty acid transporter), member 4 (Slc27a4), | 1.25745771 | 0.23948257 | 0.74302501 |
| NM_011305 | retinoid X receptor alpha (Rxra), | 1.21938205 | 0.25295311 | 0.75562513 |
| NM_008194 | glycerol kinase 1, Gyk | 1.10545289 | 0.29695401 | 0.79205298 |
| NM_009127 | Scd1 | 1.10507899 | 0.29710777 | 0.79210534 |
| NM_023114 | apolipoprotein C-III (Apoc3), | 1.08798564 | 0.30420348 | 0.79952525 |
| NM_024264 | Cyp27a1 | 1.07103403 | 0.31136955 | 0.80574574 |
| NM_021272 | Fabp7 | 1.05762477 | 0.31712977 | 0.80976612 |
| NM_172306 | cytochrome P450, family 4, subfamily a, polypeptide 12B (Cyp4a12b) | 1.02691025 | 0.3306308 | 0.82282039 |
| NM_007824 | cytochrome P450, family 7, subfamily a, polypeptide 1 (Cyp7a1), | 0.98984013 | 0.34749881 | 0.83786822 |
| NM_138648 | Olr1 | 0.94682345 | 0.36786466 | 0.85235442 |
| NM_007643 | Cd36 | 0.91836224 | 0.38180932 | 0.86377598 |
| L23108 | Cd36 | 0.91158286 | 0.3851862 | 0.86558996 |
| NM_011306 | Rxrb | 0.77576639 | 0.45730978 | 0.91539453 |
| NM_020581 | Angptl4 | 0.7360433 | 0.47999778 | 0.93067456 |
| NM_011125 | phospholipid transfer protein | 0.60516664 | 0.55965659 | 0.97966234 |
| NM_007381 | acyl-Coenzyme A dehydrogenase, long-chain (Acadl) | 0.60180387 | 0.5617991 | 0.98020551 |
| NM_183216 | stearoyl-coenzyme A desaturase 4 (Scd4), | 0.55961298 | 0.58906873 | 0.99648489 |
| NM_008256 | 3-hydroxy-3-methylglutaryl-Coenzyme A synthase 2 (Hmgcs2), | 0.53944691 | 0.60235166 | 0.99998631 |
| NM_130864 | Acaa1a | 0.45798495 | 0.65755851 | 0.99998631 |
| NM_008615 | Me1 | 0.3201504 | 0.75598577 | 0.99998631 |
| NM_011977 | Slc27a1 | 0.24655631 | 0.81064977 | 0.99998631 |
| NM_007822 | cytochrome P450, family 4, subfamily a, polypeptide 14 (Cyp4a14), | 0.20299984 | 0.8435414 | 0.99998631 |
| NM_008375 | fatty acid binding proteína 6, ileal (gastrotropin) | 0.09549713 | 0.92596198 | 0.99998631 |
| NM_013839 | nuclear receptor subfamily 1, group H, member 3 (Nr1h3), transcript variant 1 | 0.09102095 | 0.92942146 | 0.99998631 |
| NM_009948 | Cpt1b | 0.08998201 | 0.93022464 | 0.99998631 |
| NM_001033606 | Acsl3 | 0.06860339 | 0.94676913 | 0.99998631 |
| AF072757 | fatty acid transport protein 2 | 0.0471393 | 0.96340681 | 0.99998631 |
| NM_010012 | cytochrome P450, family 8, subfamily b, polypeptide 1 (Cyp8b1), | 0.04577132 | 0.96446791 | 0.99998631 |
| NM_201640 | cytochrome P450, family 4, subfamily a, polypeptide 31 (Cyp4a31) | -0.00829322 | 0.99355959 | 0.99998631 |
| NM_028994 | phosphoenolpyruvate carboxykinase 2, (Pck2) | -0.09494595 | 0.92638789 | 0.99998631 |
| NM_013474 | apolipoprotein A-II (Apoa2), | -0.13087455 | 0.89868379 | 0.99998631 |
| NM_175640 | perilipin 1, Plin | -0.13870914 | 0.89266038 | 0.99998631 |
| NM_009512 | solute carrier family 27 (fatty acid transporter), member 5 (Slc27a5) | -0.19861617 | 0.84687068 | 0.99998631 |
| NM_212444 | glycerol kinase 2, (Gyk) | -0.21357863 | 0.83552071 | 0.99998631 |
| NM_024450 | Scd3 | -0.22110372 | 0.82982739 | 0.99998631 |
| NM_009107 | retinoid X receptor gamma (Rxrg), | -0.2883475 | 0.77945377 | 0.99998631 |
| NM_019639 | EG216818, ubiquitin C (Ubc), | -0.29266757 | 0.77625144 | 0.99998631 |
| NM_007473 | aquaporin 7 (Aqp7), | -0.3819138 | 0.71117299 | 0.99998631 |
| NM_010562 | integrin linked kinase (Ilk) | -0.40595094 | 0.69403223 | 0.99998631 |
| NM_009605 | Adipoq | -0.45412121 | 0.66023584 | 0.99998631 |
| NM_007981 | acyl-CoA synthetase long-chain family member 1 (Acsl1) | -0.46891976 | 0.6500093 | 0.99998631 |
| NM_027976 | Acsl5 | -0.59206439 | 0.56803046 | 0.98474889 |
| NM_146230 | Acaa1b | -0.61601554 | 0.55277619 | 0.97496372 |
| NM_023737 | enoyl-Coenzyme A, hydratase/3-hydroxyacyl Coenzyme A dehydrogenase (Ehhadh) | -0.7159264 | 0.49175738 | 0.93769867 |
| NM_013495 | Cpt1a | -0.88705392 | 0.39758217 | 0.87456219 |
| NM_010294 | glycerol kinase 2, Gk2 | -0.90969348 | 0.38613111 | 0.8658977 |
| NM_008509 | Lpl | -1.04433516 | 0.32291882 | 0.81541816 |
| NM_080434 | apolipoprotein A-V (Apoa5), | -1.06530357 | 0.31382125 | 0.80736788 |
| NM_011144 | peroxisome proliferator activated receptor alpha | -1.31700698 | 0.21960566 | 0.72836316 |
| NM_024406 | Fabp4 | -1.4287736 | 0.18603627 | 0.70429299 |
| NM_153679 | Cpt1c | -1.52988177 | 0.1595782 | 0.70050976 |
| NM_011978 | solute carrier family 27 (fatty acid transporter), member 2 (Slc27a2), | -1.53915792 | 0.15732503 | 0.70050976 |
| NM_009949 | carnitine palmitoyltransferase 2 (Cpt2) | -1.61282268 | 0.14041198 | 0.68743949 |
| NM_032006 | matrix metallopeptidase1a Mmp1a | -1.82583656 | 0.10034642 | 0.6368321 |
| NM_015729 | acyl-Coenzyme A oxidase 1, palmitoyl (Acox1) | -1.82664372 | 0.10021702 | 0.63656188 |
| AB010100 | mRNA for aquaporin 7, complete cds | -2.02453324 | 0.07281022 | 0.595146 |
| NM_011327 | sterol carrier protein 2, liver (Scp2), | -2.1190196 | 0.06239596 | 0.58048172 |
| NM_010174 | fatty acid binding protein 3, muscle and heart (Fabp3) | -2.37746117 | 0.04075281 | 0.56515678 |
| NM_011146 | Pparg | -6.93483799 | 6.10E-010 | 0.19886968 |

**Table S2 - Table of primers and probes sequences used in RT-PCR validation.**

**Syber-Green**

**Target gene**

**Primer sequences 5´-3´**

MVK

Forward

TCCAGCAAGGGACGATGTC

Reverse

CTTGGTGTTGGTGAGCAGGAT

FADS3

Forward

AGCACCACCTCTTCCCCACGA

Reverse

CCCCAGCAGGGGGTGAGGAT

FADS2

Forward

GCGAGTGGCAGCCCCTTGAG

Reverse

GGCCCAAGCCAAGTCCACCC

TGFβ1

Forward

GCAACATGTGGAACTCTACCAG

Reverse

CAGCCACTCAGGCGTATCA

ABCA1

Forward

CGAGGCTCCCGGTGTTG

Reverse

GGCTGTACAGAAGAAGCCTCTGA

Granuphilin

Forward

CGGGACACCATCAATCCACTAT

Reverse

TGAGCCAGAAGAGATTCCGAAA

NOTCH2

Forward

GATCACCCGAATGGCTATGAAT

Reverse

CCATGGTGGCAAGGATCTG

Ndufab1

Forward

GCGCAGGTGCCTGGAA

Reverse

TCAAGACATACAGAACTCGGTCCTT

SMAD3

Forward

TCCATTCCCGAGAACACTAACTTC

Reverse

CGTCCATGCTGTGGTTCATC

NGN3

Forward

CATCCAGCTCTTGGCCCATA

Reverse

GAAGCTCAGAAACCCTGCATGT

**TaqMan**

**Target gene**

**Primer sequences 5´-3´**

18s

Forward

CGGCTACCACATCCAAGGAA

Reverse

GTCGGAATTACCGCGGCT

Probe

GAGGGCAAGTCTGGTGCCAG

PPARα

Forward

GATTCAGAAGAAGAACCGGAACA

Reverse

GCGAATTGCATTGTGTGACAT

Probe

TGCCGTTTTCACAAGTGCCTGTCTGTC

PPARδ

Forward

TGTGTGGAGACCGGCCA

Reverse

CGCAGAATGGTGTCCTGGA

Probe

GCCTCATGAATGTGCCCCAGGTAGAA

NEGR1

Forward

TGCAAGTCCCACCGAAAATAT

Reverse

GGCCAAACAAGTAAGGGTGACA

Probe

CAAATGACATGACCATCAATGAAGGAACCA

SLC30A8

Forward

TGGTCACTGACTGTGAACCAAGT

Reverse

TGCTGAGGGCTTGAGCAATT

Probe

TGTTCATGTTGCTACAGCTGCCAGCC
